# Outcomes after SRS and ipilimumab plus nivolumab for melanoma brain metastases following prior immune checkpoint inhibitor or targeted therapy

**DOI:** 10.1093/oncolo/oyag043

**Published:** 2026-02-16

**Authors:** Ian Messing, Lauren Linkowski, Matthew D Riina, Melanie Berger, Jonathan Baron, Xingmei Wang, Michael Kallan, Harper G Hubbeling, Robert A Lustig, Jay F Dorsey, Goldie Kurtz, Suneel Nagda, John N Lukens, Ahron Flowers, John Y K Lee, Nduka M Amankulor, Christina Jackson, Suyash Mohan, Ravi K Amaravadi, Lynn M Schuchter, Alexander C Huang, Tara C Mitchell, Michelle Alonso-Basanta, Emily S Lebow

**Affiliations:** Department of Radiation Oncology, University of Pennsylvania, Philadelphia, PA 19104, United States; Department of Radiation Oncology, University of Pennsylvania, Philadelphia, PA 19104, United States; Department of Radiation Oncology, University of Pennsylvania, Philadelphia, PA 19104, United States; Department of Radiation Oncology, University of Pennsylvania, Philadelphia, PA 19104, United States; Department of Radiation Oncology, University of Pennsylvania, Philadelphia, PA 19104, United States; Department of Radiation Oncology, University of Pennsylvania, Philadelphia, PA 19104, United States; Department of Radiation Oncology, University of Pennsylvania, Philadelphia, PA 19104, United States; Department of Radiation Oncology, University of Pennsylvania, Philadelphia, PA 19104, United States; Department of Radiation Oncology, University of Pennsylvania, Philadelphia, PA 19104, United States; Department of Radiation Oncology, University of Pennsylvania, Philadelphia, PA 19104, United States; Department of Radiation Oncology, University of Pennsylvania, Philadelphia, PA 19104, United States; Department of Radiation Oncology, University of Pennsylvania, Philadelphia, PA 19104, United States; Department of Radiation Oncology, University of Pennsylvania, Philadelphia, PA 19104, United States; Department of Medicine, University of Pennsylvania, Philadelphia, PA 19104, United States; Department of Neurosurgery, University of Pennsylvania, Philadelphia, PA 19104, United States; Department of Neurosurgery, University of Pennsylvania, Philadelphia, PA 19104, United States; Department of Neurosurgery, University of Pennsylvania, Philadelphia, PA 19104, United States; Department of Radiology, University of Pennsylvania, Philadelphia, PA 19104, United States; Department of Medicine, University of Pennsylvania, Philadelphia, PA 19104, United States; Department of Medicine, University of Pennsylvania, Philadelphia, PA 19104, United States; Department of Medicine, University of Pennsylvania, Philadelphia, PA 19104, United States; Department of Medicine, University of Pennsylvania, Philadelphia, PA 19104, United States; Department of Radiation Oncology, University of Pennsylvania, Philadelphia, PA 19104, United States; Department of Radiation Oncology, University of Pennsylvania, Philadelphia, PA 19104, United States

**Keywords:** melanoma brain metastases, stereotactic radiosurgery, dual immune checkpoint inhibition, ipilimumab and nivolumab, prior immune checkpoint inhibitor exposure, intracranial disease control

## Abstract

**Background:**

Melanoma brain metastases (BM) carry high morbidity and mortality despite advances in systemic therapy. Combined immune checkpoint inhibition (ICI) with ipilimumab and nivolumab (ipi/nivo) demonstrates intracranial activity, but the influence of prior systemic therapy exposure is poorly defined. This is the first real-world study evaluating outcomes of melanoma BM treated with stereotactic radiosurgery (SRS) and concurrent ipi/nivo, focusing on the impact of prior ICI or targeted therapy.

**Patients and Methods:**

We retrospectively analyzed 68 patients with 413 melanoma BM treated with concurrent SRS and ipi/nivo from 2015 to 2025. Primary endpoints were overall survival (OS) and intracranial progression-free survival (iPFS). Secondary endpoints included local and distant control, radionecrosis, and leptomeningeal disease. Univariable and multivariable Cox models identified predictors of outcome.

**Results:**

Median OS was 24.0 months (12- and 24-month OS: 64% and 50%). ICI-naive patients had longer OS (50.5 vs. 17.6 months; *P* = 0.007) and iPFS (15.1 vs. 5.9 months) than those with prior ICI. On multivariable analysis, prior ICI (HR 2.23, 95% confidence interval [CI] 1.13-4.41), prior BRAF/MEKi (HR 2.26, 95% CI 1.01-5.04), and ≥11 SRS-treated lesions (HR 3.22, 95% CI 1.43-7.21) predicted worse outcomes, while higher graded prognostic assessment (GPA) favored OS (HR 0.46, 95% CI 0.29-0.75). At 24 months, local progression was 11%, distant 49%, radionecrosis 7%, and leptomeningeal disease 4%.

**Conclusion:**

Concurrent SRS with ipi/nivo provides durable intracranial control with low toxicity. Patients with prior ICI or targeted therapy represent a high-risk subgroup with poorer outcomes, supporting exploration of intensified or novel strategies.

Implications for Practice This real-world study shows that concurrent stereotactic radiosurgery and combined ipilimumab and nivolumab achieves durable intracranial control with low toxicity for patients with melanoma brain metastases. Importantly, prior exposure to immune checkpoint inhibitors or targeted therapy identifies a high-risk subgroup with poorer outcomes. These findings emphasize the need for personalized treatment strategies, careful sequencing of systemic and local therapies, and consideration of intensified or novel approaches to optimize survival, minimize neurologic complications, and guide clinical decision-making in this vulnerable patient population.

## Introduction

Patients with advanced melanoma are at high risk of brain metastases (BM).^1^ Up to 40% of patients with metastatic melanoma have BM at diagnosis, and 60% of patients with metastatic melanoma develop BM later in their disease course.^2^ Patients with melanoma BM are at a high risk of morbidity and neurologic death.^3^^,^^4^ Survival after a diagnosis of melanoma BM remains poor,^5^^,^^6^ particularly among patients with symptomatic BM or leptomeningeal disease.^7^^,^^8^

Immune checkpoint inhibition (ICI) has revolutionized treatment for patients with metastatic melanoma. Combined ICI with ipilimumab and nivolumab (ipi/nivo) is the preferred first-line therapy among treatment naïve patients with metastatic melanoma. This recommendation is based on the Phase III CheckMate 067 trial, which showed 10-year overall survival (OS) of 52% with combined ipi/nivo.^9–11^ This represents a dramatic improvement in prognosis for patients with metastatic melanoma, with historic rates of 5-year OS of just 10%.^12^ The combination of ipi/nivo has also demonstrated intracranial activity including an intracranial response between 46% and 57% among patients with asymptomatic untreated BM.^13^^,^^14^

While ipi/nivo shows encouraging activity, there are subsets of patients less likely to have durable intracranial disease control. These include patients with symptomatic disease.^7^^,^^8^ In addition, prior systemic therapy exposure influences intracranial activity of ipi/nivo. The ABC trial showed that patients with prior exposure to BRAF/MEK inhibition (BRAF/MEKi) had worse outcomes including survival, intracranial progression-free survival (iPFS), and intracranial response rate.^8^ Retrospective analyses suggest that patients with prior ICI exposure have worse intracranial activity with ipi/nivo.^15^ ICI is increasingly used in the neoadjuvant setting, increasing the proportion of patients with melanoma BM who have had prior ICI exposure.^16^

Stereotactic radiosurgery (SRS) is a highly effective local therapy for patients with BM.^17^ This form of focal radiation delivers conformal, high doses of radiation targeting the BM with limited dose to surrounding brain. SRS is preferred to whole-brain radiation therapy (WBRT) due to reduction in cognitive toxicity.^18^ SRS can safely be delivered even for patients with numerous intracranial metastases.^19^ Prior to ipi/nivo, patients with melanoma BM received SRS or WBRT due to poor intracranial activity of systemic therapy.^20^ Recent ASCO-SNO-ASTRO national treatment guidelines allow for omission of upfront SRS among select patients with melanoma who are treatment naïve, have small, asymptomatic BM, and are planned for ipi/nivo.^21^

We evaluated outcomes among patients with metastatic melanoma treated with ipi/nivo and SRS with or without prior checkpoint exposure.

## Methods

### Study design

We retrospectively evaluated patients with melanoma BM treated with SRS and ipi/nivo at the University of Pennsylvania between November 1, 2015, and January 31, 2025. Eligible patients had pathologically confirmed metastatic melanoma and one or more BM treated with SRS and concurrent ipi/nivo, defined as at least 1 dose within 8 weeks of SRS, prior to clinical or radiographic CNS progression. The Institutional Review Board approved the study, and reporting followed STROBE guidelines.

Post–treatment brain MRI scans and patient records were manually reviewed to assess demographics, treatment history, disease control, and toxicity. Local failure was defined as progression within the prescription isodose line, and distant failure as new progression outside the prescription isodose line. Radiation necrosis was distinguished from local progression using advanced imaging (MRI perfusion and permeability imaging as well as MR Spectroscopy) and serial follow-up MRI scans. Extracranial disease was assessed using PET and CT scan of the chest, abdomen, and pelvis. Melanoma graded prognostic assessment (GPA) scores were calculated using clinically validated criteria based on established prognostic factors.^6^

Primary endpoints were OS and iPFS. Secondary endpoints included CNS progression subtype (local, distant, or both), first progression site (intracranial vs. extracranial), time to salvage radiotherapy or surgery, and incidence of symptomatic radiation necrosis and leptomeningeal disease. Time-to-event analyses were measured from the date of SRS.

### Statistical analysis

Baseline characteristics were summarized by treatment group using descriptive statistics. Group comparisons used the Wilcoxon rank-sum test or *t*-test for continuous variables and chi-squared or Fisher’s exact tests for categorical variables. OS and iPFS were estimated nonparametrically. Cumulative incidence functions, with death as a competing risk, were calculated for CNS, local, and distant progression. Clinically relevant factors were assessed using Cox proportional hazards models. Multivariable models included key clinical predictors and variables significant in univariable analyses. Analyses were performed for the overall cohort and by metastasis size (≥1 cm vs. <1 cm). Patients without events were censored at last follow-up. Statistical significance was defined as *P* < 0.05. Analyses were conducted in SAS v9.4.

## Results

We identified 68 consecutive patients with 413 BM treated with SRS and concurrent ipi/nivo at our institution (Figure S1—see online supplementary material for a color version of this figure). Patient and treatment characteristics are summarized in Table 1. Median age was 66 years; most patients were male (49, 72%), initially diagnosed with non–metastatic disease (52, 77%), harbored a BRAF mutation (36, 53%), and had extracranial disease at SRS (46, 68%).

**Table 1:** Baseline clinical characteristics.

Variable	Overall	Prior ICI	No prior ICI	*P*-value^a^
	(*N* = 68)	(*N* = 34)	(*N* = 34)	
**Median age at diagnosis**	66 (33-86)	66 (36-85)	66.5 (33-86)	0.9246
**Sex, no. (%)**				
** Male**	49 (72.1%)	24 (70.6%)	25 (73.5%)	0.7870
** Female**	19 (27.9%)	10 (29.4%)	9 (26.5%)	
**Stage at diagnosis, no. (%)**				
** I-III**	52 (76.5%)	26 (76.5%)	26 (76.5%)	1.0000
** IV**	16 (23.5%)	8 (23.5%)	8 (23.5%)	
**Performance status**				
** 100-90**	36 (52.9%)	18 (52.9%)	18 (52.9%)	1.0000
** 80**	26 (38.2%)	13 (38.2%)	13 (38.2%)	
** 70**	6 (8.8%)	3 (8.8%)	3 (8.8%)	
**Median melanoma graded prognostic assessment**	2 (0.5-3.5)	2 (0.5-3.5)	2 (0.5-3.5)	0.2145
**Melanoma graded prognostic assessment**				
** 3.5-4**	2 (2.9%)	1 (2.9%)	1 (2.9%)	0.2489
** 2.5-3**	21 (30.9%)	14 (41.2%)	7 (20.6%)	
** 1.5-2**	34 (50.0%)	14 (41.2%)	20 (58.8%)	
** 0-1**	11 (16.2%)	5 (14.7%)	6 (17.6%)	
**Extracranial metastases, no. (%)**				
** No**	22 (32.4%)	15 (44.1%)	7 (20.6%)	0.0381
** Yes**	46 (67.6%)	19 (55.9%)	27 (79.4%)	
**Median number of treated BM**	4 (1-40)	4 (1-40)	5 (1-20)	0.8328
**Number of treated BM, no. (%)**				
** 1-2**	20 (29.4%)	11 (32.4%)	9 (26.5%)	0.8991
** 3-5**	21 (30.9%)	11 (32.4%)	10 (29.4%)	
** 6-10**	16 (23.5%)	7 (20.6%)	9 (26.5%)	
** ≥11**	11 (16.2%)	5 (14.7%)	6 (17.6%)	
**Median BM diameter (mm)**	19	19	21.5	0.0542
**BM diameter, No. (%)**				
** <1 cm**	5 (7.4%)	4 (11.8%)	1 (2.9%)	0.3559
** ≥1 cm**	63 (92.6%)	30 (88.2%)	33 (97.1%)	
**Genetics, no. (%)**				
** Wild**	22 (32.4%)	8 (23.5%)	14 (41.2%)	0.1782
** BRAF**	36 (52.9%)	19 (55.9%)	17 (50.0%)	
** NRAS**	6 (8.8%)	5 (14.7%)	1 (2.9%)	
** NF1**	3 (4.4%)	2 (5.9%)	1 (2.9%)	
** KIT**	1 (1.5%)	0 (0.0%)	1 (2.9%)	
** GNAQ**	0 (0.0%)	0 (0.0%)	0 (0.0%)	
** GNA11**	0 (0.0%)	0 (0.0%)	0 (0.0%)	
**BRAF, no. (%)**				
** V600E mutant**	21 (58.3%)	13 (68.4%)	8 (47.1%)	0.5147
** V600K/R mutant**	10 (27.8%)	4 (21.1%)	6 (35.3%)	
** Other mutation**	5 (13.9%)	2 (10.5%)	3 (17.6%)	
** NA**	32 (47.1%)	15 (44.1%)	17 (50.0%)	
**Prior BRAF/MEKi therapy, no. (%)**				
** No**	55 (80.9%)	22 (64.7%)	33 (97.1%)	0.0013
** Yes**	13 (19.1%)	12 (35.3%)	1 (2.9%)	
**Prior chemotherapy, no. (%)**				
** No**	64 (94.1%)	31 (91.2%)	33 (97.1%)	0.6136
** Yes**	4 (5.9%)	3 (8.8%)	1 (2.9%)	
**Total lines of previous systemic therapy, no. (%)**				
** 0**	32 (47.1%)	0 (0.0%)	32 (94.1%)	<.0001
** 1**	24 (35.3%)	22 (64.7%)	2 (5.9%)	
** 2**	10 (14.7%)	10 (29.4%)	0 (0.0%)	
** 3**	2 (2.9%)	2 (5.9%)	0 (0.0%)	
**Neurosurgical resection, no. (%)**				
** No**	37 (54.4%)	22 (64.7%)	15 (44.1%)	0.0883
** Yes**	31 (45.6%)	12 (35.3%)	19 (55.9%)	
**Neurologic symptoms before treatment, no. (%)**				
** No**	23 (33.8%)	15 (44.1%)	8 (23.5%)	0.0728
** Yes**	45 (66.2%)	19 (55.9%)	26 (76.5%)	
**Seizures before treatment, no. (%)**				
** No**	57 (83.8%)	34 (100.0%)	23 (67.6%)	0.0004
** Yes**	11 (16.2%)	0 (0.0%)	11 (32.4%)	
**Receipt of dexamethasone, no. (%)** ^b^				
** No**	39 (57.4%)	21 (61.8%)	18 (52.9%)	0.4620
** Yes**	29 (42.6%)	13 (38.2%)	16 (47.1%)	
**Ipi/nivo cycles, no. (%)**				
** 1**	14 (20.6%)	5 (14.7%)	9 (26.5%)	0.4585
** 2**	17 (25.0%)	11 (32.4%)	6 (17.6%)	
** 3**	10 (14.7%)	5 (14.7%)	5 (14.7%)	
** 4**	27 (39.7%)	13 (38.2%)	14 (41.2%)	
**Nivo maintenance cycles, no. (%)**				
** 0-5**	40 (58.8%)	23 (67.6%)	17 (50.0%)	0.4891
** 6-10**	12 (17.6%)	4 (11.8%)	8 (23.5%)	
** 11-15**	6 (8.8%)	3 (8.8%)	3 (8.8%)	
** ≥16**	10 (14.7%)	4 (11.8%)	6 (17.6%)	
**IRAE, no. (%)** ^c^				
** No**	30 (44.1%)	13 (38.2%)	17 (50.0%)	0.3286
** Yes**	38 (55.9%)	21 (61.8%)	17 (50.0%)	

Abbreviations: BM, brain metastases; ICI, immune checkpoint inhibition.

a *P*-value is chi-square test or Fisher’s exact test for categorical variables, and Wilcoxon Rank Sum or *t*-test for continuous variables.

b At SRS.

c Immune-related adverse event.

A total of 50% of patients had prior ICI exposure, and 19% had prior BRAF/MEKi exposure. Groups did not differ by age, BM size, number of BM, performance status, stage at diagnosis, or prior surgical resection (Table 1).

Patients with prior ICI were more likely to have received prior BRAF/MEKi (35% vs. 3%, *P* = 0.002) and to have had multiple prior lines of systemic therapy (100% vs. 6%, *P* < 0.001). In contrast, ICI-naive patients were more likely to present with extracranial metastases at the time of SRS (79% vs. 56%, *P* = 0.038) and to have experienced seizures prior to SRS (32% vs. 0%, *P* < 0.001).

**Figure 1:**
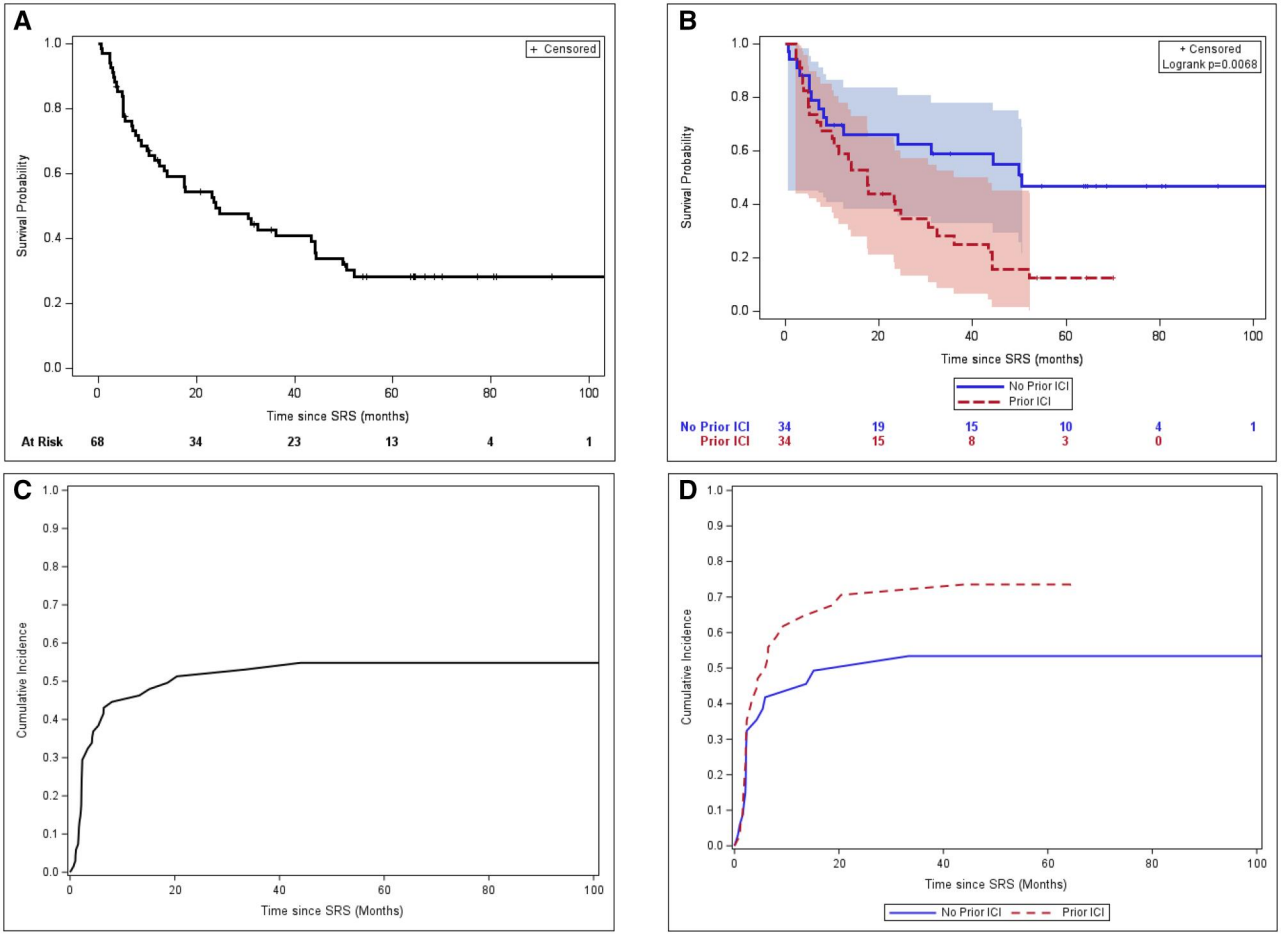
(A) Overall survival curve for all patients; (B) overall survival curve for all patients stratified by prior immune checkpoint inhibition exposure; (C) cumulative incidence of CNS progression for all patients; (D) cumulative incidence of CNS progression for all patients stratified by prior immune checkpoint inhibition exposure.

Median follow-up from SRS was 19.3 months, with a median follow-up of 63.2 months (range, 3.7-114.1 months) among patients alive at analysis. Median OS was 24.0 months, with 12- and 24-month rates of 64% and 50% (Figure 1A). Stratified by prior ICI, median OS was 50.5 months for ICI-naive patients versus 17.6 months for prior ICI (Log-rank *P* = 0.007); corresponding 12- and 24-month OS rates were 70% and 63% versus 59% and 38%, respectively (Figure 1B).

On univariable analysis (Table 2), inferior OS was associated with prior ICI (HR 2.30, 95% confidence interval [CI] 1.24-4.26, *P* = 0.008), prior BRAF/MEKi (HR 2.19, 95% CI 1.10-4.39, *P* = 0.026), and 1 (HR 2.25, 95% CI 1.13-4.46, *P* = 0.020) or 2 (HR 3.06, 95% CI 1.34-7.13, *P* = 0.008) prior systemic therapy lines; upfront surgical resection was associated with superior OS (HR 0.39, 95% CI 0.21-0.73, *P* = 0.003). Patient symptoms at presentation were not associated with OS (HR 0.65, 95% CI 0.35-1.20; *P* = 0.169). Multivariable analysis (Table 2) confirmed prior ICI (HR 2.23, 95% CI 1.13-4.41, *P* = 0.021) and prior BRAF/MEKi (HR 2.26, 95% CI 1.01-5.04, *P* = 0.047) as independent predictors of inferior OS, whereas higher GPA independently predicted superior OS (HR 0.46, 95% CI 0.29-0.75, *P* = 0.002).

**Table 2:** Univariable and multivariable analysis regression models for overall survival.

	Univariable		Multivariable	
**Characteristic**	**HR (95 CI)**	***P*-value**	**HR (95 CI)**	***P*-value**
**Age at diagnosis**	1.00 (0.97, 1.03)	0.8879		
**Sex**				
** Male**	Reference	NA		
** Female**	0.65 (0.35, 1.20)	0.1688		
**Performance status**				
** 100-90**	Reference	NA		
** 80**	1.27 (0.68, 2.37)	0.4568		
** 70**	1.87 (0.71, 4.95)	0.2068		
**BM diameter (mm)**	0.99 (0.97, 1.02)	0.7013		
**BM diameter (mm)**				
** <1 cm**	Reference	NA		
** ≥1 cm**	1.01 (0.31, 3.27)	0.9821		
**GPA**	1.00 (0.97, 1.03)	0.8879	0.46 (0.29, 0.75)	0.0019
**GPA**				
** 3.5-4**	0.41 (0.05, 3.44)	0.4131		
** 2.5-3**	0.57 (0.21, 1.52)	0.2613		
** 1.5-2**	1.20 (0.49, 2.94)	0.684		
** 0-1**	Reference	NA		
**Prior ICI**				
** No**	Reference	NA	Reference	NA
** Yes**	2.30 (1.24, 4.26)	0.0084	2.23 (1.13, 4.41)	0.0209
**Prior BRAF/MEKi Therapy**				
** No**	Reference	NA	Reference	NA
** Yes**	2.19 (1.10, 4.39)	0.0264	2.26 (1.01, 5.04)	0.0473
**Prior chemotherapy**				
** No**	Reference	NA		
** Yes**	1.37 (0.49, 3.83)	0.5487		
**Neurosurgical resection**				
** No**	Reference	NA		
** Yes**	0.39 (0.21, 0.73)	0.0031		
**Ipi/nivo cycles**	0.90 (0.72, 1.14)	0.4003		
**Number of treated BM**	1.04 (1.00, 1.08)	0.0519		
**Number of treated BM**				
** 1-2**	Reference	NA		
** 3-5**	1.15 (0.54, 2.45)	0.7237		
** 6-10**	0.89 (0.37, 2.15)	0.7957		
** ≥11**	2.73 (1.18, 6.31)	0.0184		
**Extracranial metastases**				
** No**	Reference	NA		
** Yes**	1.18 (0.64, 2.20)	0.5964		
**Symptoms at presentation**				
** No**	Reference	NA		
** Yes**	0.65 (0.35, 1.20)	0.1688		
**Seizure prior to treatment**				
** No**	Reference	NA		
** Yes**	0.52 (0.21, 1.34)	0.1761		
**Lines of prior therapy**	1.53 (1.12, 2.08)	0.0078		
**Lines of prior therapy**				
** 0**	Reference	NA		
** 1**	2.25 (1.13, 4.46)	0.0203		
** 2**	3.09 (1.34, 7.13)	0.0080		
** 3**	2.19 (0.49, 9.71)	0.3020		
**Stage at diagnosis**				
** Localized**	Reference	NA		
** Metastatic**	1.30 (0.66, 2.58)	0.4474		

Abbreviations: BM, brain metastases; GPA, graded prognostic assessment; ICI, immune checkpoint inhibition.

Cumulative incidence of CNS progression was 52% at 12 months and 61% at 24 months (Figure 1C). Stratified by prior ICI, median iPFS was 15.1 months for ICI-naive patients versus 5.9 months for prior ICI (Log-rank *P* = 0.120); corresponding 12- and 24-month CNS progression rates were 42% and 49% versus 62% and 71%, respectively (Figure 1D).

On univariable analysis (Table 3), higher CNS progression risk was associated with GPA >1.0-≤2 (HR 3.72, 95% CI 1.07-12.9, *P* = 0.039), 3 prior systemic therapy lines (HR 1.78, 95% CI 1.06-3.00, *P* = 0.028), and ≥11 lesions treated with SRS (HR 2.49, 95% CI 1.07-5.79, *P* = 0.034); older age at BM diagnosis was associated with lower risk (HR 0.97, 95% CI 0.95-0.99, *P* = 0.009). Patient symptoms at presentation were not associated with CNS progression (HR 1.10, 95% CI 0.57-2.09; *P* = 0.784). Multivariable analysis (Table 3) identified prior ICI (HR 2.08, 95% CI 1.12-3.86, *P* = 0.021), ≥11 SRS-treated lesions (HR 3.22, 95% CI 1.43-7.21, *P* = 0.005), and larger BM size (HR 1.05 per mm, 95% CI 1.01-1.08, *P* = 0.011) as independent predictors of CNS progression.

**Table 3:** Univariable and multivariable analysis competing risk regression models for CNS progression with competing risk of death.

	Univariable		Multivariable	
**Characteristic**	**HR (95 CI)**	***P*-value**	**HR (95 CI)**	***P*-value**
**Age at diagnosis**	0.97 (0.95, 0.99)	0.0091		
**Sex**				
** Male**	Reference	NA		
** Female**	0.97 (0.54, 1.75)	0.9322		
**Performance status**				
** 100-90**	Reference	NA		
** 80**	0.73 (0.36, 1.47)	0.3788		
** 70**	1.03 (0.36, 2.91)	0.9553		
**BM diameter (mm)**	1.03 (1.00, 1.06)	0.0736	1.05 (1.01, 1.08)	0.0106
**BM diameter (mm)**				
** <1 cm**	Reference	NA		
** ≥1 cm**	1.73 (0.40, 7.53)	0.4684		
**GPA**	1.00 (0.69, 1.45)	0.9959		
**GPA**				
** 3.5-4**	1.48 (0.20, 10.9)	0.6992		
** 2.5-3**	2.18 (0.62, 7.70)	0.2246		
** 1.5-2**	3.72 (1.07, 12.9)	0.0389		
** 0-1**	Reference	NA		
**Prior ICI**				
** No**	Reference	NA	Reference	NA
** Yes**	1.62 (0.88, 2.97)	0.1223	2.08 (1.12, 3.86)	0.0210
**Prior BRAF/MEKi therapy**				
** No**	Reference	NA		
** Yes**	1.06 (0.53, 2.11)	0.8702		
**Prior chemotherapy**				
** No**	Reference	NA		
** Yes**	1.07 (0.44, 2.63)	0.8811		
**Neurosurgical resection**				
** No**	Reference	NA		
** Yes**	1.02 (0.56, 1.85)	0.9463		
**Ipi/nivo cycles**	0.89 (0.70, 1.14)	0.3759		
**Number of treated BM**	1.03 (1.00, 1.06)	0.0367		
**Number of treated BM**				
** 1-2**	Reference	NA	Reference	NA
** 3-5**	1.32 (0.64, 2.73)	0.4508	1.50 (0.73, 3.08)	0.2756
** 6-10**	1.09 (0.45, 2.67)	0.8472	1.32 (0.53, 3.28)	0.5494
** ≥11**	2.49 (1.07, 5.79)	0.0337	3.22 (1.43, 7.21)	0.0046
**Extracranial metastases**				
** No**	Reference	NA		
** Yes**	0.69 (0.37, 1.27)	0.2285		
**Symptoms at presentation**				
** No**	Reference	NA		
** Yes**	1.10 (0.57, 2.09)	0.7836		
**Seizure prior to treatment**				
** No**	Reference	NA		
** Yes**	1.38 (0.68, 2.78)	0.3720		
**Lines of prior therapy**	1.27 (0.96, 1.68)	0.0952		
**Lines of prior therapy**				
** 0**	Reference	NA		
** 1**	1.63 (0.83, 3.22)	0.1593		
** 2**	1.64 (0.67, 4.04)	0.2776		
** 3**	1.78 (1.06, 3.00)	0.0283		
**Stage at diagnosis**				
** Localized**	Reference	NA		
** Metastatic**	1.53 (0.82, 2.86)	0.1793		

Abbreviations: BM, brain metastases; GPA, graded prognostic assessment; ICI, immune checkpoint inhibition.

When stratified by brain metastasis size, the cumulative incidence of CNS progression for lesions ≥1 cm was 53% at 12 months and 62% at 24 months; corresponding rates were 43% and 50% for ICI-naive patients versus 63% and 73% for those with prior ICI (Gray’s test *P* = 0.117). For lesions <1 cm, the cumulative incidence was 40% at both 12 and 24 months, with no events observed among ICI-naive patients and rates of 40% for prior ICI (Gray’s test *P* = 0.435).

**Figure 2:**
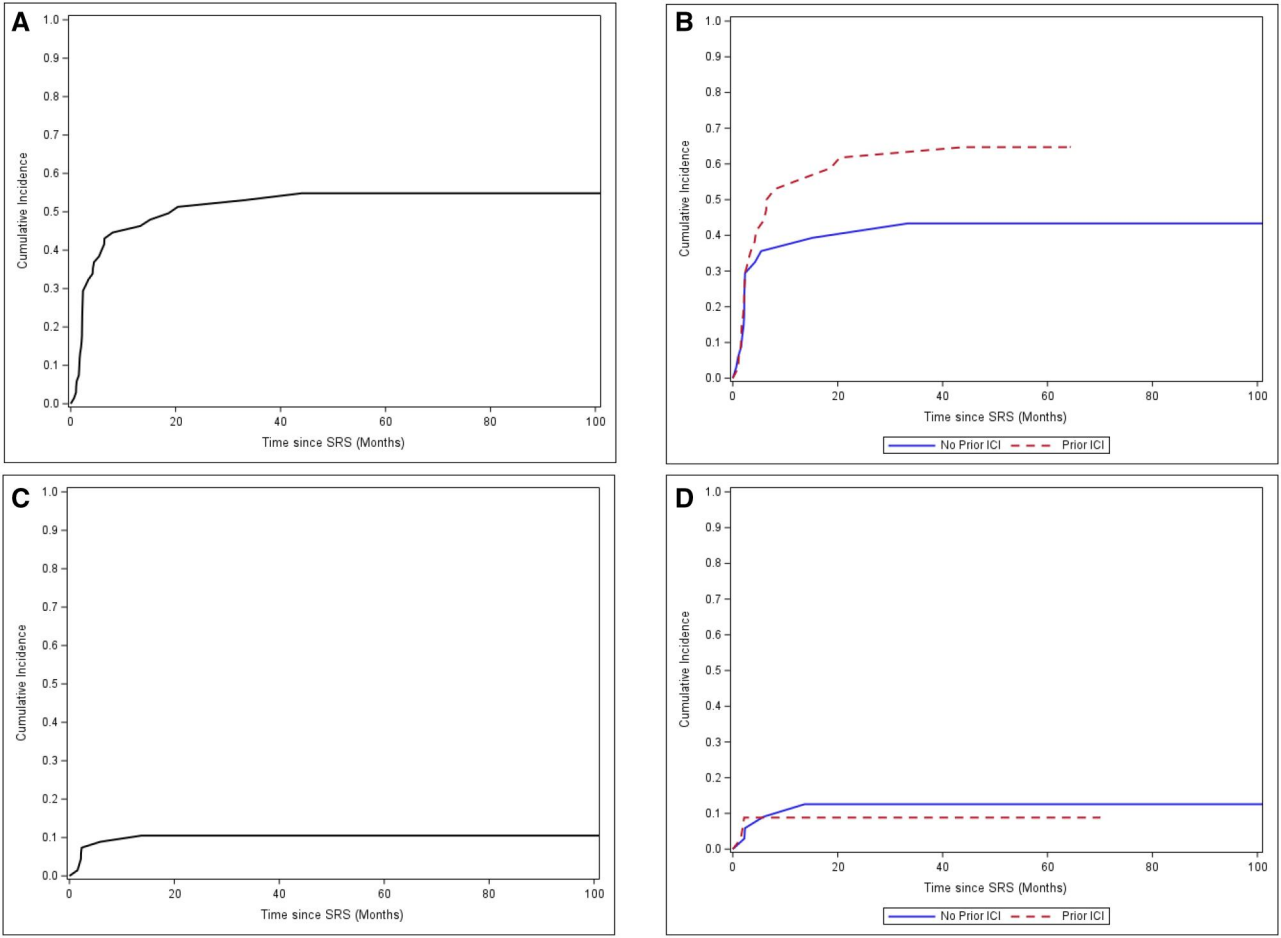
(A) Cumulative incidence of distant CNS progression for all patients; (B) cumulative incidence of distant CNS progression for all patients stratified by prior immune checkpoint inhibition exposure; (C) Cumulative incidence of local CNS progression for all patients; (D) cumulative incidence of local CNS progression for all patients stratified by prior immune checkpoint inhibition exposure.

Cumulative incidence of distant progression was 45% at 12 months and 51% at 24 months (Figure 2A). Stratified by prior ICI, 12- and 24-month distant progression rates were 36% and 39% for ICI-naive patients versus 53% and 62% for prior ICI patients (Gray’s test *P* = 0.141; Figure 2B).

Cumulative incidence of local progression was 9% at 12 months and 11% at 24 months (Figure 2C). Stratified by prior ICI, 12- and 24-month local progression rates were 9% and 13% for ICI-naive patients versus 9% and 9% for prior ICI patients (Gray’s test *P* = 0.709; Figure 2D).

No differences were observed in first progression site (*P* = 0.400) or first CNS progression (*P* = 0.086) between groups (Table 4). Similarly, rates of salvage brain radiation (*P* = 0.022) or salvage surgery (*P* = 0.742) did not differ (Table 4).

**Table 4:** Patterns of first progression and subsequent management.

Characteristic	Prior ICI (*N* = 34), No. %		No Prior ICI (*N* = 34), No. (%)		*P*-value^a^
**Site of first progression**					0.4003
** CNS progression as a component of first progression**	15	(44.1%)	12	(35.3%)	
** Extra-CNS only**	12	(35.3%)	10	(29.4%)	
** No known disease progression**	7	(20.6%)	12	(35.3%)	
**First CNS progression type**					0.0752
** Distant only**	22	(64.7%)	13	(38.2%)	
** Local progression as a component of first progression**	3	(8.8%)	4	(11.8%)	
** No CNS progression**	9	(26.5%)	17	(50.0%)	
**Salvage brain radiation**					0.2153
** No**	18	(52.9%)	23	(67.6%)	
** Yes**	16	(47.1%)	11	(32.4%)	
**Salvage brain surgery**					0.7441
** No**	29	(85.3%)	28	(82.4%)	
** Yes**	5	(14.7%)	6	(17.6%)	

Abbreviation: ICI, immune checkpoint inhibition.

a Pearson’s Chi-squared test; Fisher’s exact test.

Indications for prior ICI are summarized in Table 5. Most patients received ICI for Stage IV disease (17, 50%), with others treated for Stage I-III disease (15, 44%) or as neoadjuvant therapy (2, 6%). PD-1/PD-L1 therapy was most common (24, 71%), with CTLA-4 (3, 9%) and dual checkpoint inhibition (7, 21%) also administered. Among patients with prior ICI exposure, the median number of ICI cycles was 6. Median interval between prior ICI and concurrent SRS with ipi/nivo was 8.7 months (range, 0.5-144.7 months).

**Table 5:** Prior immune checkpoint inhibitor characteristics.

Characteristic	*N* = 34, no (%)	
**Indication for ICI**		
** Stage I-III disease**	15	(44.1%)
** Stage IV disease**	17	(50.0%)
** Neoadjuvant therapy**	2	(5.9%)
**Type of ICI**		
** PD-1/L1**	24	(70.6%)
** CTLA-4**	3	(8.8%)
** Both**	7	(20.6%)
**Median number of cycles of prior ICI**	6	
**Median months between prior ICI and Ipi/Nivo**	8.7	

Abbreviation: ICI, immune checkpoint inhibition.

Symptomatic radionecrosis and leptomeningeal disease occurred in 5 patients (7%) and 3 patients (4%), respectively.

## Discussion

In this retrospective real-world cohort of patients with melanoma BM treated with SRS and combined ipi/nivo, we observed a 2-year OS of 50% and a 60% incidence of CNS progression at 2 years, reflecting that a subset of patients are able to achieve long-term intracranial disease control and survival despite the presence of active BM. Furthermore, we observed 2-year cumulative incidence of recurrence of 11% and low rates of symptomatic toxicity, reflecting the relative safety and efficacy of this combination of therapies. These findings represent a significant advance in care for patients with melanoma BM who historically had a median survival of 5 months and were at high risk of neurologic morbidity and mortality.^2^^,^^22^ A critical challenge remains identifying biomarkers predictive of poor intracranial outcomes and survival to better guide therapy escalation for the highest risk patients.

Multiple contemporary studies have demonstrated that combining SRS with ICI is both feasible and effective in patients with melanoma BM. Studies have shown excellent 12-month local control rates exceeding 90% when SRS is delivered concurrently with nivolumab or ipilimumab and confirmed that this approach is safe, with no increase in high-grade toxicity compared with non–concurrent treatment.^23^^,^^24^ More recent analyses have found that adding SRS to dual checkpoint inhibition improves intracranial progression-free and OS compared with immunotherapy alone.^25^^,^^26^ Collectively, these findings support the safety and efficacy of SRS–ICI integration and are consistent with our results demonstrating excellent local control and a low rate of symptomatic necrosis.

Our findings build on recent landmark trials of combined ipi/nivo among patients with melanoma BM, including Checkmate-204 and the ABC trial.^7^^,^^8^ Checkmate-204 was a Phase II trial of ipi/nivo among patients with metastatic melanoma and untreated BM, reporting a 3-year OS of 72% among patients with asymptomatic BM. Of note, outcomes were significantly worse among patients with symptomatic BM with 3-year OS of 37%.^7^ The ABC trial randomized patients with untreated melanoma BM to ipi/nivo versus nivolumab alone. Among asymptomatic patients treated with ipi/nivo (Cohort A), the 7-year OS was 48%.^8^ Of note, both Checkmate-204 and the ABC trial excluded patients with prior exposure to ICI.^7^^,^^8^ Furthermore, the ABC trial prohibited enrollment of patients with prior surgical resection or large BM. Notably, half of patients in our cohort had received prior ICI, and two-thirds were symptomatic at presentation, and approximately one-third required surgical resection. These notable differences may account for the difference in survival (2-year OS of 50%) among this real-world cohort of patients.

Ongoing prospective studies, including the ABC-X trial evaluating ipi/nivo with or without upfront SRS, will further define the optimal integration and sequencing of local and systemic therapies for patients with melanoma BM.^27^

We identified prior systemic therapy disease exposures as a critical predictor of outcomes among melanoma patients with BM receiving SRS including ICI and targeted therapy. Prior retrospective analyses suggest that progression on prior ICI therapy is associated with worse intracranial outcomes. For example, a retrospective review of patients with metastatic melanoma with progressive BM after prior anti–PD-1 therapy showed an overall intracranial response rate of 11% and median intracranial PFS of 1.6 months.^15^ Receipt of prior targeted therapy was also identified as a predictor of poor outcomes in the ABC trial in which prior BRAF/MEKi was associated with worse intracranial response rates, intracranial PFS, and OS.^8^ This is consistent with other studies evaluating the sequencing of ICI and targeted therapy among patients with BRAF-mutated melanoma,^26^^,^^28^^,^^29^ including the SECOMBIT trial which showed that patients who had BRAF/MEKi followed by ICI had a shorter survival after brain metastasis diagnosis compared to patients receiving the opposite sequence of therapies.^29^ Unique disease biology among patients with progression on BRAF/MEK progression therapy likely influences response to subsequent ICI.^30^

We observed that differences in intracranial disease control among prior ICI treated patients were due to development of additional sites of intracranial disease and not due to differences in local control (Figure 2). Local control of lesions treated with SRS remained excellent with a cumulative incidence of recurrence of approximately 10% at 2 years. Furthermore, we noted a rate of symptomatic necrosis of 7% among the entire cohort which is lower than other series of SRS and checkpoint inhibition.^23^^,^^31^^,^^32^ Given the potential neurologic morbidity of poorly controlled BM, the combination of SRS and ipi/nivo remains an important therapeutic option to minimize neurologic morbidity and mortality among patients with melanoma BM, and in particular those patients with prior systemic therapy exposures, larger or symptomatic lesions, or requiring surgical resection.

Our findings also align with emerging intracranial activity data from the phase I/IIa RELATIVITY-020 trial, which evaluated nivolumab plus relatlimab in patients refractory to anti–PD-(L)1 therapy.^33^ In the CNS-specific post–hoc analysis, confirmed intracranial overall response rate was 22%, with a clinical benefit rate (partial response plus stable disease) of 63%. The median time to intracranial response was 3.2 months, and 63% of patients remained free of intracranial progression for over 3 years.^34^ Of note, the majority of patients in this analysis (81%) had prior brain radiation. These data highlight how the evolving systemic therapy landscape including emergence of new combination of ICI may alter management paradigms for patients with melanoma BM. Given that the majority of patients in this cohort received radiation, further studies will be required to elucidate the optimal integration and sequencing of dual ICI with local therapies including SRS among these high-risk subsets of melanoma patients.

Radiation therapy may potentiate ICI,^35^ including reinvigoration of a systemic response despite apparent progression.^36^ This synergy occurs through a diverse set of mechanisms^37^ including increased production of type I interferon,^38^ activation of tumor-associated dendritic cells and CD8 T-cells,^39^ and enhanced MHC-Class I expression on tumor cells.^40^ Studies to date have focused on the relationship between extracranial radiation and ICI potentiation. Additional mechanistic studies are required to understand how intracranial radiosurgery may potentiate both intracranial and extracranial ICI activity. Such studies may inform the optimal sequences of SRS and ICI among patients with BM from melanoma and other disease histologies.

This study has several limitations. Its retrospective design and single-institution cohort may limit generalizability. Selection bias and confounding by indication are possible, as patients who received prior ICI likely had more aggressive disease or treatment resistance. Subgroup analyses, particularly those by brain metastasis size and prior therapy, were limited by small sample sizes, reducing statistical power. Although median follow-up exceeded 19 months, longer-term outcomes and late toxicities may be underestimated, especially among more recently treated patients. In addition, the treatment landscape for melanoma has evolved over the study period, with greater use of neoadjuvant and adjuvant ICI and novel systemic combinations, suggesting that the effects observed in our study may be even more pronounced in current practice. Despite these limitations, our analysis provides important insights into outcomes for patients with melanoma BM receiving SRS and ipi/nivo, highlighting factors associated with inferior OS and intracranial control, particularly prior systemic therapy exposure.

## Conclusion

In conclusion, SRS combined with ipi/nivo offers excellent intracranial disease control with low risk of toxicity. However, subsets of patients including those with prior ICI or targeted therapy exposure, have worse outcomes. These findings underscore the importance of personalized management strategies and support ongoing investigation of sequencing and combination approaches for patients with BM in the evolving era of checkpoint therapy.

## Supplementary Material

oyag043_Supplementary_Data

## Data Availability

The data that support the findings of this study are not publicly available.
