# Supplementary figures and images for: Outcomes after SRS and ipilimumab plus nivolumab for melanoma brain metastases following prior immune checkpoint inhibitor or targeted therapy
Source: Oncologist. 2026 Feb 16;31(4):oyag043. doi: 10.1093/oncolo/oyag043 (PMC13010309; doi:10.1093/oncolo/oyag043)

Supplementary Figure 1. CONSORT Diagram

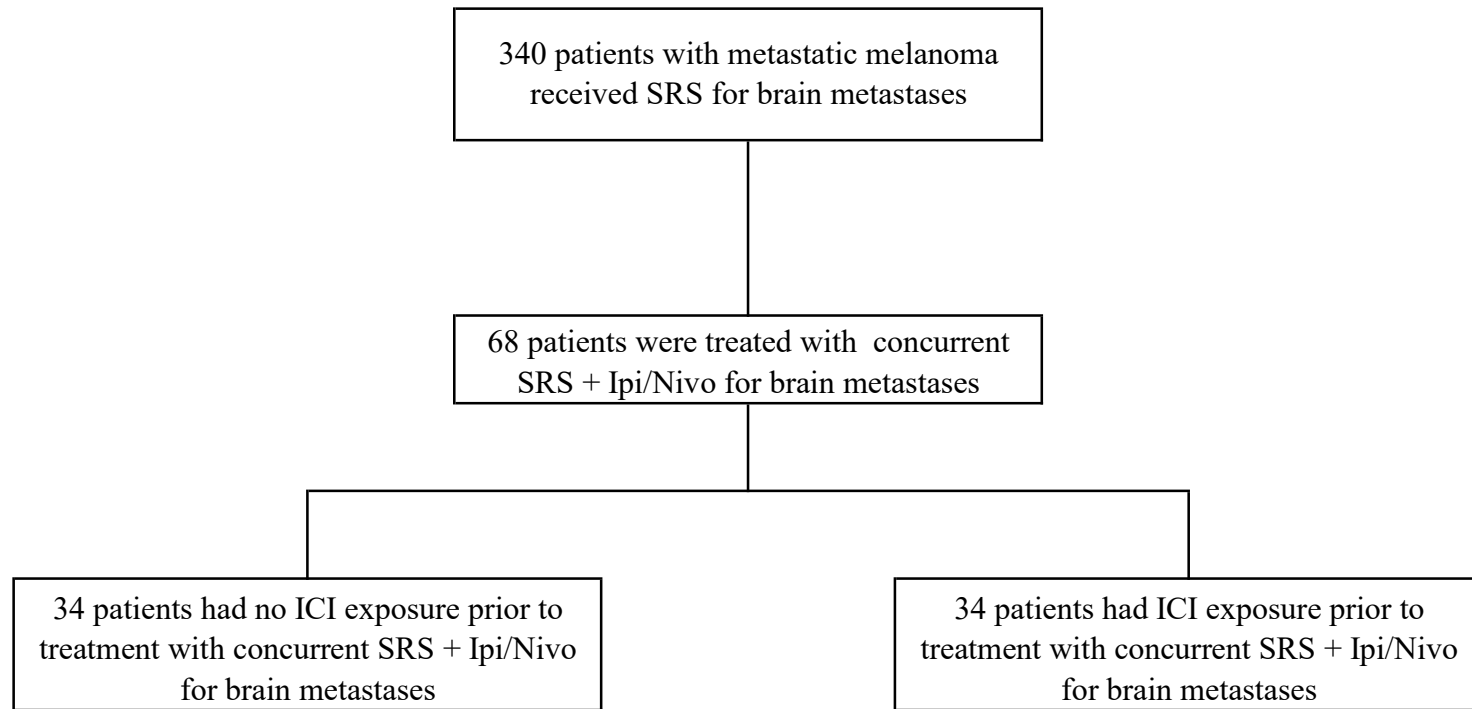

Supplement: oyag043_Supplementary_Data [file oyag043_supplementary_data.pdf]
